# Urinalysis and Urinary GGT-to-Urinary Creatinine Ratio in Dogs with Acute Pancreatitis

**DOI:** 10.3390/vetsci6010027

**Published:** 2019-03-13

**Authors:** Eleonora Gori, Alessio Pierini, Ilaria Lippi, Noemi Boffa, Francesca Perondi, Veronica Marchetti

**Affiliations:** Department of Veterinary Sciences, University of Pisa, Via Livornese Lato Monte, 56121 Pisa, Italy; pierini.alessio2004@gmail.com (A.P.); ilariausa@gmail.com (I.L.); noe.boffa@gmail.com (N.B.); f.perondi87@gmail.com (F.P.); veronica.marchetti@unipi.it (V.M.)

**Keywords:** urinalysis, dog, acute pancreatitis, kidney injury, urinary GGT-to-creatinine ratio, prognosis

## Abstract

In acute pancreatitis (AP), kidney injury (KI) can occur. Urinalysis and some urinary biomarkers have been proposed as prognostic tools in human AP. The aim of the study was to evaluate urinalysis and urinary GGT-to-urinary creatinine (uGGT/uCr) in canine AP and their association with possible outcomes. AP diagnosis was based on clinical and laboratory parameters, abnormal SNAP^®^ cPL™ test and compatible imaging. Urinary KI (uKI) was defined if dogs had urinary casts and/or proteinuria. Dogs (n = 70) were divided in survivors and non-survivors according to the 15-day outcome. Data were analyzed using statistical software. Seventy dogs were retrospectively included, of which 24 dogs (34%) died. uKI was detected in 36 dogs (37%) which was associated with mortality (*p* = 0.01, Odds ratio (OR) 3.9, 95% CI 1.3–11.56). Non-survivors showed higher dipstick bilirubin levels than survivors (*p* = 0.0022). By excluding active sediments, urine protein-to-creatinine ratio (UPC) ≥2 was associated with mortality (*p* = 0.001, OR 47.5, 95% CI 4–571.9). The uGGT/uCr was available in 40 dogs, although no association of this factor with any outcome was found. The UPC ≥2 can be a negative prognostic factor in canine AP and further studies on uGGT/uCr are warranted.

## 1. Introduction

Acute pancreatitis (AP) is the most common disease of the exocrine pancreas in dogs, with a very variable clinical presentation [1]. Fortunately, most dogs show mild to moderate clinical signs that are characterized by various degrees of vomiting, lethargy, inappetence, abdominal pain, diarrhea [1,2,3]. More severe forms of AP are characterized by the development of a systemic inflammatory response syndrome (SIRS) [4]. 

During AP-induced SIRS, multiple organ impairment (MODS) can occur as the result of a “cytokine storm” that can damage more sensible organs and apparatus, especially kidneys and lungs [4,5,6]. The AP diagnosis can be made based upon clinical presentation, hematobiochemical profile (acute inflammation parameters), diagnostic imaging (abdominal ultrasound) and specific tests (canine pancreas-specific lipase; cPL) [1,6,7]. To evaluate the renal impairment during AP in dogs, as well as the evaluation of serum urea and creatinine [6], urinalysis can help the clinician in the overview of renal function. 

During AP, kidney injury (KI) can occur via hypovolemia, cytokine-induced ischemia, inflammation and oxidative stress [5]. In people, urinalysis and some urinary biomarkers have been proposed as useful prognostic tools in AP [8]. Urinary GGT-to-urinary creatinine ratio (uGGT/uCr) has been evaluated in various studies as a urinary KI (uKI) marker [9,10] as it seems to be a good marker for the detection of acute kidney injury (AKI), especially as a tubular damage marker [11].

To the best of the authors’ knowledge, there have been no previous studies that evaluated urinalysis in canine AP. The aim of the study was to evaluate urinalysis parameters and urinary GGT-to-urinary creatinine ratio (uGGT/uCr) in canine AP and to determine their association with possible outcomes.

## 2. Materials and Methods

Dogs with AP who had been hospitalized at the Veterinary Teaching Hospital between September 2016 and January 2018 were identified from the hospital management system. Since each owner signs an informed consent to allow to use of dogs’ medical records in our veterinary teaching hospital and this study involved a retrospective research in medical records, a formal ethical approval was not necessary.

The diagnosis of AP was based on (1) the acute onset of two or more of the following clinical signs: abdominal pain, diarrhea, vomiting or anorexia/hyporexia; (2) abdominal ultrasound (Xario XG, Toshiba, Tokyo, Japan) suggestive of AP without other identifiable extra-pancreatic diseases; and (3) abnormal SNAP® cPL test result (Idexx Laboratories, Milan, Italy). 

Abdominal ultrasound was considered to be compatible with AP diagnosis if there were the following ultrasonographic changes: hypoechoic areas within the pancreatic parenchyma, hyperechoic mesenteric areas surrounding the pancreas, various degrees of pancreas enlargement and abdominal effusion [1]. Dogs with clinical signs and clinicopathological features compatible with AP but without a positive abdominal ultrasound at hospital admission were rechecked every 24 h and included if they developed ultrasonographic findings compatible with AP, within 48 h from their admission. 

Dogs with a history of renal diseases (clinical records/history, bloodwork and diagnostic imaging), urinary tract infection and/or on hemodialysis treatment were excluded, along with dogs with acute abdomen of non-pancreatic origin, and dogs that had received known nephrotoxic drugs (e.g., non-steroidal anti-inflammatory drugs, aminoglycosides).

In the veterinary teaching hospital, urine samples are collected and analyzed within 12 h of the collection (IDEXX VetLab UA Analyzer and Idexx UA Strips, Idexx, Milan, Italy). Urine protein-to-creatinine ratio (UPC) and uGGT/uCr are routinely performed in the hospital using a biochemistry analyzer (Liasys, Assel SRL, Rome, Italy). For uGGT/uCr, a cut-off value of 105 U/g was used [11]. uKI was defined if dogs had urinary casts and/or proteinuria. Sediments were classified as active if there were one or more of the following findings: bacteriuria and >5 RBCs, WBCs, or epithelial cells/hpf. Dogs were divided into two groups (survivors and non-survivors) according to 15-day outcome from the hospital admission. Normal distribution was assessed using D’Agostino–Pearson test. Urine specific gravity (USG), UPC and uGGT/uCr were evaluated in association with the outcome using the Mann–Whitney U-test. pH was compared between outcome groups using an unpaired *t*-test. A chi square test was used to evaluate dipstick parameters in association with the outcome. Fisher’s exact test was used to compare the severity of UPC (≥2 or <2) and the presence of uKI to the outcome. Odds ratio (OR) was also calculated. Data were analyzed using a commercial statistical software package (Graphpad Prism 7 for Mac OS X, GraphPad Software Inc, La Jolla, CA, USA).

## 3. Results

A total of 70 client-owned dogs with owners’ informed consent fulfilled the inclusion criteria. The median age of the dogs was 9.8 years (range 0.7–16.5 years). There were 19 mixed breed dogs. The most represented breeds were: Beagle and German Shepherd dogs (five dogs each), Labrador Retrievers (four dogs), Poodle, Dobermann Pinscher, Lagotto Romagnolo (three dogs each), Great Dane, Cocker Spaniel, French Bulldog, Rottweiler, Hound, English Setter, English Springer Spaniel (two dogs each) and one each of the following: Daschund, Border Collie, Boxer, Bouvier des Flandres, German Shorthaired Pointer, Épagneul Breton, English bulldog, Pug, Flat Coated Retriever, Maltese, Maremma Shepherd, German Pinscher, Siberian Husky, Golden Retriever, Jack Russell Terrier, Yorkshire Terrier. Thirty-three dogs (47%) were females, eight of them intact, and the remaining 37 dogs (53%) were males, six of them neutered.

**Figure 1:**
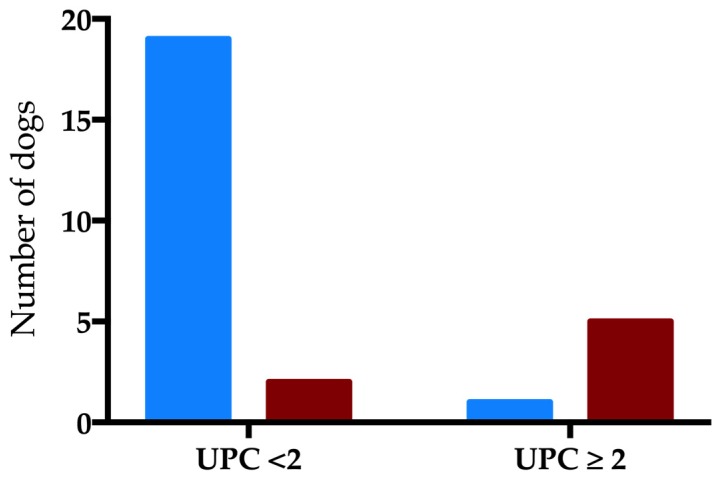
Evaluation of urine protein-to-creatinine ratio (UPC) severity in the outcome groups (blue pattern: survivors; red pattern: non-survivors) (*p* = 0.001).

**Figure 2:**
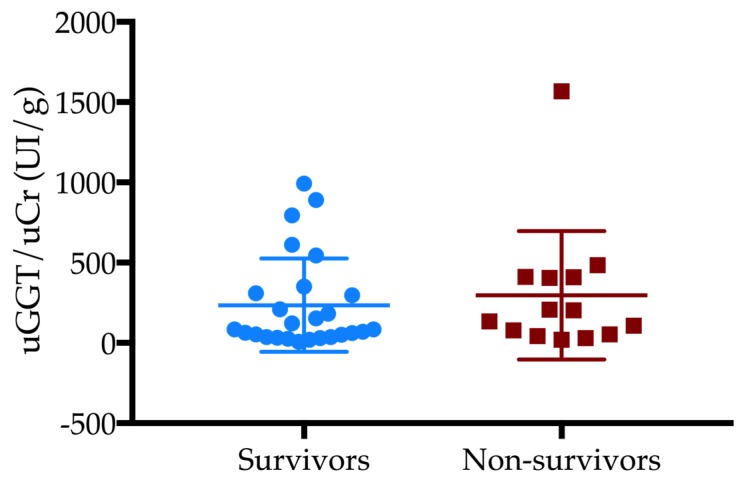
Urinary GGT-to-urinary creatinine (uGGT/Cr) values between survivors and non-survivors (*p* > 0.05).

Twenty-four dogs (34%) died. Seven out of 24 dogs were euthanized due to a worsening in clinical condition, or to a progression of the disease. Urine samples were collected by free catch (n = 43), cystocentesis (n = 19) or catheterization (n = 8). Forty dogs showed active sediment (57%). uKI was detected in thirty-six dogs (37%) and was associated with mortality (*p* = 0.01, OR 3.9, 95% CI 1.3–11.56). Non-survivor dogs showed higher dipstick bilirubin levels compared to the surviving dogs (*p* = 0.005). SG, pH and the other dipstick parameters were similar between groups (Table 1). Non-survivor dogs showed higher values of UPC compared to survivor dogs (*p* = 0.0087). By excluding dogs with active sediment (n = 40), UPC and its severity (UPC ≥2) were associated with mortality (*p* = 0.03 and *p* = 0.001, OR 47.5, 95% CI 4–571.9, respectively; Figure 1). uGGT/uCr was available in 40 dogs (57%). Twenty-one dogs (53%) had uGGT/uCr over the cut-off level which was not associated with the outcome. No statistical differences were found in uGGT/uCr values between survivor and non-survivor dogs (Figure 2).

## 4. Discussion

As far as we are aware, during AP, various classes of cytokines (“cytokine storm”), especially interleukin-1, -2, -6 and tumor necrosis factor alfa, and reactive oxygen species can cause multiple organ impairment, especially in the kidneys and lungs [4]. 

In both human and veterinary medicine, there is constant research conducted on early biomarkers that can predict renal impairment and acute kidney injury (AKI). In humans, the development of AKI is responsible for 70–80% of early deaths in patients with AP [5,12,13,14]. For this reason, predicting the severity and understanding the prognosis of these patients is of crucial importance. In dogs with AP, serum creatinine has been proposed as a negative prognostic marker, as well as the presence of AKI [6,15]. Unfortunately, serum creatinine levels have limited value as an early marker of renal impairment, as it only changes when the majority of the renal mass is damaged [16]. 

To date, this is the first study of an evaluation of urinalysis abnormalities and uGGT/uCr in dogs with AP. In this study, more than the half of the population showed an active sediment. This finding could be due to the high prevalence of free catches in our sampling method, compared to cystocentesis. In fact, voided samples of urine may be contaminated with cells and bacteria located in the distal urethra, genital tract, and/or on the skin and hair [17]. 

High urinary bilirubin concentrations were associated with mortality. Increased bilirubinuria may be present in human patients with AP [8]. The renal threshold for bilirubin is very low in dogs, therefore small amounts of bilirubin may be present in the urine of healthy dogs, even when serum bilirubin is normal [18]. During AP, bilirubinuria could be caused by some subclinical hemolytic processes or possible disseminated intravascular coagulation due to systemic inflammation and also as a result of an impairment of a bile duct outflow [1,8]. On the other hand, increased urine bilirubin may be observed during AP-induced hepatic injury [1,8]. However, only three non-survivor dogs showed mild hyperbilirubinemia at the biochemical panel.

In the present study, non-survivors showed higher values of UPC than survivors. We evaluated the UPC excluding active sediment in order to avoid bias in the interpretation of this result. In addition, a study on the comparison of UPC using cystocentesis versus free catch in dogs showed how the UPC was minimally affected by free catch, thus allowing the correct grading of proteinuria with this method [19]. A human paper on urinalysis in humans demonstrated how high urinary protein and low specific gravity were associated with more severe acute disease and with the development of AKI [8]. To the best of our knowledge, there is only one veterinary study evaluating renal impairment in dogs with AP, which showed how high UPC was associated with this outcome [15]. Urinary kidney injury (uKI) was defined as the presence of casts and/or proteinuria. Casts originate in the kidney, and their presence supports a diagnosis of renal impairment, as well as the presence of proteinuria [18]. 

We failed to find an association between uGGT/uCr and the outcome. Urinary GGT is located in the metabolically active portion of the proximal tubule. It can be found in the urine as a consequence of acute tubular injury [9,10]. Recently, Lippi et al. [11] studied uGGT/uCr in healthy dogs and in dogs with various renal diseases (AKI, stable chronic kidney disease (CKD), lower urinary tract infections). In the latter study, the authors showed how GGT/uCr could be a potentially good marker for the diagnosis of AKI because dogs with AKI showed significantly higher levels of uGGT/uCr as compared to healthy dogs [11]. In azotemic dogs, uGGT/uCr may increase as a consequence of a reduced renal creatinine clearance. The healthy dogs showed a maximum value of uGGT/uCr of 105.6 UI/g and for this reason we chose this value as the threshold for our dogs. In our study, more than half of the dogs had a uGGT/uCr value over the chosen cut-off level (>105 U/g), proving that, even if uGGT/uCr was not associated with the outcome, a mild-to-moderate non-fatal tubular damage during AP could occur.

This study has several limitations. First of all, there was not a standardization in the urine sampling method. Cystocentesis may be the best way to prevent urine contamination with bacteria, cells, etc. from the lower urogenital tract [17]. Secondly, dogs may have had other diseases besides AP that could have affected urinalysis, such as endocrinopathies and hepatopathies. The authors could not rule out CKD stage 1 dogs, due to the lack of glomerular filtration rate (GFR) or Symmetric dimethylarginine (SDMA) evaluation. We were only able to collect and analyze 40 urine samples for the evaluation of uGGT/uCr. Increasing the number of samples should provide more interesting results, especially including a basal value and a follow-up level in the urine. Although histopathology is still considered the gold standard for the differentiation of acute from chronic pancreatitis, this investigation is not feasible in clinical practice. To date, a lack of histopathology in AP diagnosis has been replaced by using multiple diagnostic investigations as specific tests for pancreatic specific lipase such as through a hematobiochemical profile and diagnostic imaging exams.

Urinalysis is a cheap and feasible exam that could help the clinician to assess renal impairment. In dogs with AP kidney injury it is fairly common and seems to be a negative prognostic factor. Proteinuria, during AP, resulted in a greater negative prognostic marker in urinalysis. Further research needs to include large-scale prospective studies of the significance and trends associated with uGGT/uCr in canine AP. 

## Figures and Tables

**Table 1:** Evaluation of dipstick parameters between survivors and non-survivors.

Parameter	Survivors (n = 46)	Non-Survivors (n = 24)	*p*-Values
pH *	6.5 ± 1	6.3 ± 0.9	0.35
**SG ^§^**	1018 (1007–1050)	1017 (1004–1048)	0.42
**PRO**			
neg	30 (43%)	10 (14%)	0.09
TR	0 (0%)	0 (0%)	
1+	8 (11%)	6 (9%)	
2+	5 (7%)	2 (3%)	
3+	3 (4%)	6 (9%)	
**GLU**			
neg	39 (56%)	24 (34%)	0.24
1+	2 (3%)	0 (0%)	
2+	1 (1%)	0 (0%)	
3+	0 (0%)	0 (0%)	
4+	4 (6%)	0 (0%)	
**KET**			
neg	38 (54%)	20 (29%)	0.3
1+	5 (7%)	4 (6%)	
2+	3 (4%)	0 (0%)	
3+	0 (0%)	0 (0%)	
**UBG**			
norm	16 (24%)	6 (9%)	0.23
1+	24 (35%)	11 (15%)	
2+	5 (7%)	3 (4%)	
3+	1 (1%)	3 (4%)	
4+	0 (0%)	1 (1%)	
**BIL**			
neg	34 (49%)	15 (22%)	**0.022**
1+	3 (4%)	1 (1%)	
2+	8 (11%)	2 (3%)	
3+	1 (1%)	6 (9%)	
**BLD/HGB**			
neg	23 (33%)	7 (10%)	0.17
1+	3 (4%)	0 (0%)	
2+	6 (9%)	4 (6%)	
3+	4 (6%)	2 (3%)	
4+	10 (14%)	11 (15%)	

SG, specific gravity; PRO, protein; GLU, glucose; KET, ketones; UBG, urobilinogen; BIL, bilirubin; BLD/HGB, blood/hemoglobin. * *p*-value obtained with unpaired t-test. § p-value obtained using a Mann–Whitney U-test. All the other p-values were obtained using a chi square test.
